# Clinical outcomes following endometrial receptivity assessment-guided personalized euploid embryo transfer in patients with previous implantation failures

**DOI:** 10.1038/s41598-025-01056-5

**Published:** 2025-05-15

**Authors:** María Ruiz-Alonso, Carlos Gómez, Tiffany Stankewicz, José A. Castellón, Antonio Díez-Juan, Eva Gómez, Carmen Rubio, Carlos Simón, Diana Valbuena

**Affiliations:** 1 Igenomix SL, Parque Tecnológico de Paterna, Ronda Narciso Monturiol Estarriol 11B, Paterna, Valencia, 46980 Spain; 2 Department of Pediatrics, Obstetrics & Gynecology, University of Valencia, Valencia, 46010 Spain; 3 Department of Obstetrics and Gynecology, Beth Israel Deaconess Medical Center, Harvard Medical School, Boston, MA 02215 USA; 4 Carlos Simon Foundation, Parque Tecnológico de Paterna, Ronda Narciso Monturiol Estarriol 11C, Paterna, Valencia, 46980 Spain

**Keywords:** Embryonic implantation, Window of implantation, Implantation failure, Endometrial receptivity analysis, Personalized embryo transfer, Euploid blastocyst, Infertility, Transcriptomics

## Abstract

This study aimed to investigate potential improvements when implementing endometrial receptivity analysis (ERA)-guided personalized embryo transfer (pET) using euploid blastocyst in patients with one or more previous failed embryo transfers. A total of 270 patients with one or more previous failed embryo transfers were enrolled between 2017 and 2021 in this multicenter retrospective study. These patients were divided into two groups: study cases (ERA-guided pET) (*n* = 200) or controls (standard embryo transfer) (*n* = 70). Clinical outcomes compared were pregnancy rate (PR), implantation rate (IR), ongoing pregnancy rate (OPR), and live birth rate (LBR). Clinical results in terms of PR, OPR, and LBR were significantly higher when performing ERA-guided pET (PR: 65.0%; OPR: 49.0%; LBR: 48.2%) compared to standard embryo transfer (PR: 37.1%; OPR: 27.1%; LBR: 26.1%) (*P* < 0.01). Logistic regression was applied to examine the correlation between ERA and the primary outcome measured, OPR, including demographic variables as covariates. The effect of ERA was significantly associated with OPR (*P* = 0.002; aOR 2.8, 95% CI 1.5–5.5); furthermore, OPR decreased significantly when body mass index (BMI) values increased (*P* = 0.04; aOR 0.9, 95% CI 0.8–0.98). These findings support the potential of ERA-guided pET to improve clinical outcomes and address the challenges encountered by patients with previous implantation failures.

**Supplementary Information:**

The online version contains supplementary material available at 10.1038/s41598-025-01056-5.

## Introduction

While *Homo sapiens *have evolved to boast high levels of intelligence, our fertility rates have not kept pace. A systematic review published in 2022 estimated that the infertility rate in reproductive-aged couples could reach 17.5%^1^. The window of implantation (WOI), spanning 30–36 h, marks the critical period when the maternal endometrium becomes receptive to blastocyst implantation. This window typically occurs six to eight days after the luteinizing hormone (LH) surge in natural cycles or four to seven days following endogenous or exogenous progesterone exposure^2–4^.

During the 2000´s, research focusing on bulk endometrial transcriptomics identified gene expression profiles related to the different phases of the menstrual cycle. Four independent groups simultaneously reported the transcriptomic profiling of the secretory phase of the human endometrium during natural cycles, searching for the WOI^5–8^. Two additional groups extended this transcriptomic characterization across the menstrual cycle^9,10^. Subsequent studies expanded their scope to include controlled ovarian stimulation (COS) cycles^11–13^ and even refractory cycles in patients with inert intrauterine devices (IUD)^14^.

The pioneering transcriptomics-based “Endometrial Receptivity Analysis” (ERA) test was designed to diagnose endometrial receptivity status in infertile patients experiencing implantation failure originating from the endometrium^15^. The ERA test enables personalized embryo transfer (pET) by aligning the individualized WOI of each patient with the developmental stage of their blastocyst. In addition, single-cell RNA sequencing-based research has validated this comprehensive transcriptomics dataset^16^.

Unfortunately, the results of ERA-guided pET remain contentious after over a decade of clinical application. Thus far, 43 publications containing varying levels of evidence have meticulously scrutinized the clinical outcomes of ERA-guided pET across specific clinical indications and geographical locations, leading to divergent conclusions (Supplementary Table 1 provides comprehensive coverage of published studies).

Notably, four recent meta-analyses - considered the pinnacle of the evidence-based pyramid – have been published. Three of the four suggest the potential utility of assessing endometrial receptivity in patients experiencing repeated implantation failure (RIF) of endometrial origin^17–19^, while a study by Arian^20^ suggested no benefit. Recently, findings from the first randomized controlled trial evaluating the ERA test in patients with RIF supported the clinical benefit of euploid pET^21^.

Since first appearing in the early 1990´s, PGT-A has become a critical tool in any in vitro fertilization (IVF) center, helping professionals to increase pregnancy rates and decrease miscarriage rates^22^. Nevertheless, the transfer of a euploid embryo does not always lead to the achievement of a pregnancy owing to the implication of additional factors, such as the endometrial factor. Thus, combining PGT-A and the ERA test may provide an important clinical benefit to IVF patients. Multiple studies sought to explore the potential benefits of this approach, with reported results both for^21,23–26^ and against^27–31^ the implementation of this combination. Of note, most of these studies involved RIF or first appointment population, with low representation of patients with just 1–2 previous implantation failures, which is where the scientific evidence remains limited.

**Table 1:** Demographics and clinical characteristics of patients with and without personalized embryo transfer. Values presented as mean ± sd or %. Wilcoxon rank-sum test applied to quantitative comparisons. Categorical variables compared using the Chi-square or fisher exact test. P-values indicate the statistical significance of differences between groups. Body-mass index (BMI) represents weight in kilograms divided by the square of the height in meters. “Optimal” and “suboptimal” categories used for embryo quality assessment (blastocyst quality not available for all the embryos). Abbreviations – ERA: endometrial receptivity analysis; ET: embryo transfer; pET: personalized embryo transfer; PGT-A: preimplantation genetic testing for aneuploidy; SD: standard deviation; WOI: window of implantation.

PGT-A patients	ERA-guided pET	Standard ET	*P*-value
Number of patients	200	70	-
Mean age ± SD	35.3 ± 3.9	35.8 ± 3.3	0.31
Mean BMI ± SD	23.0 ± 3.0	23.3 ± 3.7	0.61
Mean previous failed attempts ± SD	2.4 ± 1.8	2.2 ± 1.8	0.32
Mean number of MII oocytes ± SD	11.7 ± 7.1	11.9 ± 7.7	0.83
Mean number of fertilized oocytes ± SD	9.3 ± 5.7	9.4 ± 4.5	0.89
Mean number of vitrified embryos ± SD	4.4 ± 2.8	4.5 ± 2.6	0.84
Mean blastocysts transferred ± SD	1.1 ± 0.3	1.1 ± 0.4	0.79
Single embryo transfer (%)	177/200 (88.5)	62/70 (88.6)	> 0.9
Double embryo transfer (%)	23/200 (11.5)	8/70 (11.4)	> 0.9
Blastocysts with optimal quality (%)	138/203 (68.0)	42/71 (59.2)	0.23
Blastocysts with suboptimal quality (%)	65/203 (32.0)	29/71 (40.8)	0.23
Patients with displaced WOI (%)	83/200 (41.5)	-	-

This study aims to explore an intermediate perspective by assessing the clinical outcomes of ERA-guided pET in patients who have experienced at least one failed embryo transfer and underwent preimplantation genetic testing for aneuploidy (PGT-A).

## Materials and methods

### Study design

This is a multicenter retrospective study comparing ERA-guided pET versus standard embryo transfer, both with euploid blastocysts, in patients with one or more previous failed embryo transfers.

### Patient population

This study involves 270 patients with at least one previous failed embryo transfer performed between 2017 and 2021. Data were exported from our electronic health database and anonymized in compliance with specific legislation on biomedical research, personal data protection, and bioethics. The study was evaluated and approved by the independent Scientific Committee and the Ethical Committee of the Hospital Clínico Universitario de Valencia (approval No. 2021/018) and it was performed in accordance with relevant guidelines and regulations. The consent form waiver for this retrospective study was also approved by the independent Scientific Committee and the Ethical Committee of the Hospital Clínico Universitario de Valencia. Patients were divided into two groups: the study group underwent ERA-guided, euploid pET (*n* = 200), and the control group underwent standard euploid embryo transfer (*n*= 70). The transfer of euploid embryos assures the lack of chromosomal abnormalities in embryos as a potential cause of implantation failure or miscarriage. This study reports one embryo transfer per patient, considering the first embryo transfer after performing the ERA test in the pET group. All embryo transfers for both groups were performed in hormone replacement therapy (HRT) cycles. Single and double embryo transfers (SET and DET, respectively) were considered in this study. Variables such as age, body mass index (BMI), previous failed attempts, number of metaphase II (MII) and fertilized oocytes, number of vitrified embryos and transferred embryo quality were compared between the two groups. Embryo quality was evaluated at the inner cell mass and trophectoderm levels according to Gardner’s criteria^32^, classifying the embryos as optimal (categories A + B) or suboptimal (categories C + D).

### ERA endometrial biopsy, analysis, and interpretation of results

An endometrial biopsy for ERA was obtained during an HRT cycle. The type and dosage of progesterone varied slightly depending on the clinic’s standard of care. For example, a typical HRT cycle involves estradiol priming (delivered orally [6 mg daily] or via patches [two patches every two days]) on the first or second day of the menstrual cycle. Ultrasound assessment is performed seven to ten days after estradiol priming starts. Progesterone intake starts when a trilaminar endometrium > 6 mm is reached with serum progesterone levels of < 1 ng/ml (within 24 h before exogenous progesterone starts). Progesterone is administered for five full days (approximately 120 h), with 400 mg of micronized vaginal progesterone given every 12 h (800 mg per day). Overall, the first intake of progesterone occurs on day *P* + 0, with endometrial biopsies obtained on day *P* + 5.

The endometrial biopsy isolates a small uterine lining sample at the fundus. A pipette is inserted through the vagina and cervix into the uterus, where a small piece of tissue is extracted.

The ERA molecular diagnostic tool utilizes next-generation sequencing (NGS) to analyze the expression levels of 248 genes related to endometrial receptivity status. The ERA computational predictor then identifies the specific transcriptomic signatures for each endometrial stage: proliferative, pre-receptive, receptive, late receptive, and post-receptive. A pET recommendation is then offered based on this result, indicating the optimal moment of receptivity during the menstrual cycle. For patients with a receptive result, pET is recommended following the same conditions and timing in which the ERA biopsy was collected. For patients who received a “pre-receptive” result, pET is recommended a specified number of hours/days later in the cycle in relation to the time the ERA biopsy was collected. For patients who received a “post-receptive” or a “late receptive” result, pET is recommended at a specified number of hours/days earlier in relation to the time the ERA biopsy was collected.

### Embryo transfer and clinical outcomes

After identifying the patient’s WOI, ERA-guided pET was performed for patients in the ERA group. Participants in the control group underwent standard embryo transfer. Transfers of euploid embryos were performed in HRT cycles in all cases for both groups.

Clinical outcomes compared between both groups were pregnancy rate (PR), implantation rate (IR), ongoing pregnancy rate (OPR), and live birth rate (LBR). PR was calculated as the percentage of all patients positive for free beta human chorionic gonadotrophin (βhCG); IR was defined as the number of gestational sacs observed by vaginal ultrasound at the fifth gestational week divided by the total number of embryos transferred; OPR was calculated as the percentage of pregnancies that continued beyond the 12 th gestational week for all embryo transfers; and LBR was calculated as the number of deliveries that resulted in at least one live birth per embryo transfer from the total of patients receiving an embryo transfer with completed follow-up until delivery.

### Statistical analyses

The Wilcoxon rank-sum test was applied to quantitative comparisons. Categorical variables were expressed in percentages and compared using Chi-square or Fisher exact tests. Multivariate logistic regression was used to evaluate the association between ERA and OPR. Maternal age, BMI, previous failed attempts, number of embryos transferred, and embryo quality were covariates to calculate the adjusted odds ratio (aOR). The corresponding confidence intervals were also computed for each coefficient. Statistical analysis was performed using statistical software R (version 4.2). p values *≤* 0.05 were considered statistically different.

## Results

In total, 270 patients were enrolled in this study: 200 in the ERA-guided pET group and 70 in the standard euploid embryo transfer (control) group. Among the pET group, 117 patients displayed a receptive result (58.5%), while 83 exhibited a displaced WOI (41.5%), with 74 (89.2%) being pre-receptive, 6 (7.2%) late receptive and 3 (3.6%) post-receptive. We compared demographic variables, including age, BMI, number of previous failed embryo transfers, number of MII oocytes, number of fertilized oocytes, number of vitrified embryos, and the number of embryos transferred, and morphological analyses of embryo quality of transferred blastocysts between groups (Table 1). These comparisons revealed no significant differences between the pET and control groups.

We observed significant differences in clinical outcomes when comparing patients undergoing ERA-guided pET versus standard embryo transfer of euploid blastocysts. The PR reached 65.0% in the pET group, significantly surpassing the 37.1% observed in the control (*P* < 0.001). Additionally, we observed a significantly higher OPR in the pET group (49.0%) compared to control (27.1%) (*P* = 0.002). We obtained follow-up information until delivery in 197/200 patients in the pET group and 69/70 patients in the control group, revealing a significantly higher LBR in the pET group (48.2%) compared to the control group (26.1%) (*P* = 0.002). The remaining early pregnancy loss rates (clinical miscarriages, biochemical and ectopic pregnancy rates) remained similar in both groups (Table 2).

**Table 2:** Clinical outcomes of patients with and without personalized embryo transfer. Data expressed as %. Variables compared using the Chi-square or fisher exact test. P-values indicate the statistical significance of differences between groups. Abbreviations – ERA: endometrial receptivity analysis; ET: embryo transfer; pET: personalized embryo transfer; PGT-A: preimplantation genetic testing for aneuploidy.

PGT-A patients	ERA-guided pET	Standard ET	*P*-value
Number of patients	200	70	-
Implantation rate (%)	125/223 (58.0)	23/79 (32.9)	< 0.001
Pregnancy rate (%)	130/200 (65.0)	26/70 (37.1)	< 0.001
Biochemical pregnancy rate (%)	13/130 (10.0)	3/26 (11.5)	0.73
Clinical miscarriage rate (%)	18/130 (13.9)	4/26 (15.4)	0.76
Ectopic pregnancy rate (%)	1/130 (0.8)	0	0.99
Ongoing pregnancy rate (%)	98/200 (49.0)	19/70 (27.1)	0.002
Lost to follow-up rate (%)	3/200 (1.5)	1/70 (1.4)	0.99
Live birth rate (%)	95/197 (48.2)	18/69 (26.1)	0.002

A sub-analysis including just cycles with SET provided a similar trend, with significant differences for the pET group compared to the control group (Supplementary Table 2).

We next conducted a subclassification based on the number of previous failed embryo transfers (one or two or more previous failed embryo transfers) (Table 3). For those cases with one previous failed embryo transfer, the comparison revealed a higher IR (56.6% versus 28.6%; *P* = 0.01) and PR (65.7% versus 35.3%; *P* = 0.007) when applying ERA. Furthermore, we found a significantly higher OPR (47.1% versus 23.5%; *P* = 0.04) and LBR (46.4% versus 23.5%; *P* = 0.04) in the pET group.

**Table 3:** Demographics, clinical characteristics, and outcomes according to the number of previous failed embryo transfers. Data expressed as mean ± sd and %. Wilcoxon rank-sum test applied to quantitative comparisons. Categorical variables compared using the Chi-square or fisher exact test. P-values indicate the statistical significance of differences between groups. Different subscripts (a or b) report statistically significant differences between the equal previous number of failed embryo transfers across patient groups. Body-mass index (BMI) represents weight in kilograms divided by the square of the height in meters. The number of embryos transferred, single embryo transfer, double embryo transfer, and blastocyst quality refer to the study of embryo transfer. Abbreviations – ERA: endometrial receptivity analysis; ET: embryo transfer; pET: personalized embryo transfer; SD: standard deviation.

Previous failed ET	ERA-guided pET		Standard ET	
	1	*≥* 2	1	*≥* 2
Number of patients	70	130	34	36
Age (mean ± SD)	35.1 *±* 3.8	35.4 *±* 3.9	35.8 *±* 3.9	35.7 *±* 2.8
BMI (mean ± SD)	22.6 *±* 3.0	23.2 *±* 3.0	22.5 *±* 3.0	24.0 *±* 4.2
*Number of MII oocytes* *(mean ± SD)*	12.3 *± 6.8*	11.4 *± 7.4*	12.7 *±* 7.5	11.2 *±* 7.9
*Number of fertilized oocytes (mean ± SD)*	9.9 ± 5.7	8.9 ± 5.8	9.5 ± 4.4	9.3 ± 7.7
*Number of vitrified embryos* *(mean ± SD)*	4.6 *±* 2.8	4.3 ± 2.8	5.1 *±* 2.6	3.8 *±* 2.3
Number of embryos transferred (mean *±* SD)	76 (1.1 *±* 0.3)	147 (1.1 *±* 0.3)	35 (1.0 *±* 0.2)	44 (1.2 *±* 0.5)
Single embryo transfer (%)	64/70 (91.4)	113/130 (86.9)	33/34 (97.1)	29/36 (80.6)
Double embryo transfer (%)	6/70 (8.6)	17/130 (13.1)	1/34 (2.9)	7/36 (19.4)
Blastocysts with optimal quality (%)	56/68 (82.4)	82/135 (60.7)	26/33 (78.8)	16/38 (42.1)
Blastocysts with suboptimal quality (%)	12/68 (17.6)	53/135 (39.3)	7/33 (21.2)	22/38 (57.9)
Implantation rate (%)	$\:43\text{/}76{\left(56.6\right)}^{a}$	$\:82\text{/}147{\left(55.8\right)}^{b}$	$\:10\text{/}35{\left(28.6\right)}^{a}$	$\:13\text{/}44{\left(29.6\right)}^{b}$
Pregnancy rate (%)	$\:46\text{/}70{\left(65.7\right)}^{a}$	$\:84\text{/}130{\left(64.6\right)}^{b}$	$\:12\text{/}34{\left(35.3\text{\%}\right)}^{a}$	$\:14\text{/}36{\left(38.9\right)}^{b}$
Biochemical pregnancy rate (%)	5/46 (10.9)	8/84 (9.5)	2/12 (16.7)	1/14 (7.1)
Clinical miscarriage rate (%)	8/46 (17.2)	10/84 (11.9)	2/12 (16.7)	2/14 (14.3)
Ectopic pregnancy rate (%)	0	1/84 (1.2)	0	0
Ongoing pregnancy rate (%)	$\:33\text{/}70{\left(47.1\right)}^{a}$	65/130 (50.0)	$\:8\text{/}34{\left(23.5\right)}^{a}$	11/36 (30.6)
Lost to follow-up rate (%)	1/70 (1.4)	2/130 (1.54)	0	1/36 (2.8)
Live birth rate (%)	$\:32\text{/}69{\left(46.4\right)}^{a}$	$\:63\text{/}128{\left(49.2\right)}^{b}$	$\:8\text{/}34{\left(23.5\right)}^{a}$	$\:10\text{/}35{\left(28.6\right)}^{b}$

In cases with two or more previous failed embryo transfers, we found a significantly higher IR in the pET group versus the control group (55.8% versus 29.6%; *P* = 0.004) (Table 3) and a significantly higher PR in the pET group versus the control group (64.6% versus 38.9%; *P* = 0.01). For the OPR, we observed a similar trend in favor of the pET group as for cases with one previous failed embryo transfer; however, this difference failed to reach significance due to the smaller sample size (50.0% versus 30.6%; *P* = 0.06) (Table 3). Finally, we found a significantly higher LBR in the pET group compared to the control group (49.2% versus 28.6%; *P* = 0.047) (Table 3).

We also compared clinical outcomes in the pET group between patients with standard and displaced WOIs. We did not encounter significant differences in the pET group, as transfers were performed according to their personalized WOI (Table 4).

**Table 4:** Demographics, clinical characteristics, and outcomes according to window of implantation (WOI) displacement. Data expressed as mean ± sd and %. Wilcoxon rank-sum test applied to quantitative comparisons. Categorical variables compared using the Chi-square or fisher exact test. P-values indicate the statistical significance of differences between groups. Body-mass index (BMI) represents weight in kilograms divided by the square of the height in meters. The number of embryos transferred, single embryo transfer, double embryo transfer, and blastocyst quality refer to the study of embryo transfer. Abbreviations SD: standard deviation.

WOI	Displaced	Non-Displaced	*P*-value
Number of patients	83	117	
Age (mean ± SD)	35.4 *±* 3.9	35.2 *±* 3.9	0.68
BMI (mean ± SD)	22.9 *±* 2.8	23.1 *±* 3.2	0.72
Previous failed attempts (mean ± SD)	2.3 *±* 1.7	2.5 *±* 1.9	0.42
Number of MII oocytes (mean ± SD)	11.6 ± 6.0	11.8 ± 7.7	0.87
Number of vitrified oocytes (mean ± SD)	9.1 ± 4.9	9.4 ± 6.3	0.65
Number of vitrified embryos (mean ± SD)	4.5 ± 2.7	4.3 ± 2.9	0.56
Number of embryos transferred (mean ± SD)	93 (1.1 ± 0.3)	130 (1.1 ± 0.3)	0.84
Single embryo transfer (%)	73/83 (88.0)	104/117 (88.9)	> 0.9
Double embryo transfer (%)	10/83 (12.0)	13/117 (11.1)	> 0.9
Blastocysts with optimal quality (%)	57/84 (67.9)	81/119 (68.1)	0.99
Blastocysts with suboptimal quality (%)	27/84 (32.1)	38/119 (31.9)	0.99
Implantation rate (%)	52/93 (55.9)	73/130 (56.2)	0.99
Pregnancy rate (%)	53/83 (63.9)	77/117 (65.8)	0.89
Biochemical pregnancy rate (%)	5/53 (9.4)	8/77 (10.4)	0.99
Clinical miscarriages rate (%)	10/53 (18.9)	8/77 (10.4)	0.26
Ectopic pregnancy rate (%)	0	1/77 (1.3)	0.99
Ongoing pregnancy rate (%)	38/83 (45.8)	60/117 (51.3)	0.53
Lost to follow-up rate (%)	2/83 (2.41)	1/117 (0.85)	0.57
Live birth rate (%)	36/81 (44.4)	59/116 (50.9)	0.46

Finally, we undertook a multivariant binomial logistic regression to study the relationship between ERA-guided pET and the principal studied variable (OPR), considering interfering control variables. This analysis revealed that ERA significantly influenced the OPR (*P* = 0.002; aOR 2.8, 95% CI 1.5–5.5) (Supplementary Table 3). Additionally, we discovered that the BMI significantly impacted the OPR (*P* = 0.04; aOR 0.9, 95% CI 0.8–0.98) (Supplementary Table 3).

## Discussion

The WOI represents a specific time frame during the menstrual cycle when the endometrium allows embryo implantation; therefore, understanding and accurately pinpointing the WOI remains important for a successful embryo transfer. Endogenous factors play a significant role in shaping the WOI, with hormonal imbalances, irregular menstrual cycles, high BMI, or conditions like polycystic ovary syndrome known as disrupting factors^33–36^.

An extensive body of research has revealed the greater prevalence of implantation difficulties in certain groups, such as RIF patients or older individuals^37,38^. RIF - a challenging condition associated with multiple unsuccessful embryo transfers - could arise due to issues related to the WOI; therefore, determining the timing of endometrial receptivity and employing ERA-guided pET could represent an important means of improving implantation^2,39^.

Our study reveals that ERA-guided pET supports a significantly higher PR, OPR, and LBR than standard embryo transfer (65.0% versus 37.1%, 49.0% versus 27.1%, and 48.2% versus 26.1%, respectively). These results provide evidence that ERA-guided pET notably enhances clinical outcomes, specifically in cases involving patients with previous implantation failures when the use of PGT-A rules out the embryo factor. Furthermore, multivariate testing revealed a relationship between BMI and OPR in PGT-A patients, underlining the relevance of female weight to outcomes when minimizing the impact of the embryo factor^35,36^. Obese women suffer from worse results and a high number of miscarriages that negatively affect LBR after euploid embryo transfer^40–42^. These data reinforce the importance of endometrial factors on reproductive outcomes, given the relationship between BMI and the WOI^36,43^.

Subclassification based on the number of previous failed embryo transfers (one or two or more) further demonstrated improved clinical outcomes with ERA-guided pET compared to standard ET, revealing that the ERA effectively addressed cases of implantation failure (Table 3). A deeper analysis of the pET group revealed that the correction in patients with a displaced WOI supported clinical results similar to those without WOI displacement.

Of note, the gradual nature of the changes in the endometrial transcriptome during the menstrual cycle means that not all cases of WOI displacement have equal relevance at the clinical level. The possibly frequent occurrence of an endometrium existing in a transition between pre-receptivity and receptivity would make the adjustment of progesterone exposure timing irrelevant in a healthy population with a normal hormonal balance, regular menstrual cycles, and no prior implantation failures. This has been addressed in some studies^20 27^, raising fundamental questions regarding the appropriate application of ERA-guided pET in assisted reproductive technologies. However, most of these studies were focused on good prognosis patients, where the benefit of ERA is less evident. In contrast, recent studies, including one randomized controlled trial and recent meta-analyses, have shown the potential benefits of ERA-guided pET in patients with RIF^2,21,39^. Thus, selecting patients with previous failed embryo transfers may serve as a filter to select clinically relevant WOI displacements; employing ERA-guided pET in these cases can make a significant difference, as observed in the present study and other studies reporting similar findings^2,39^.

Considering the evolving technological and clinical landscape, these findings warrant further prospective controlled trials to confirm and generalize our findings. These prospective studies would support the comprehensive investigation of other factors influencing implantation success to ensure a holistic approach to improving fertility treatment outcomes and to refine the criteria for selecting the most appropriate transfer method for each patient.

While this analysis depends on pre-existing data and suffers from a risk of bias due to the study’s retrospective nature, our findings offer a solid foundation for prospective future research.

## Conclusion

In this study we have found that 41.5% of patients from the ERA-guided pET group presented WOI displacement. These patients showed similar clinical outcomes to those with a non-displaced WOI, after WOI correction. Furthermore, results have shown that ERA-guided pET on euploid embryos improved clinical outcomes in patients with at least one previous failure attempt. These results support that ERA-guided pET has the potential to enhance clinical outcomes, demonstrating chances in tackling the difficulties faced by patients with previous implantation failure These findings underscore the potential of personalized medicine to increase reproductive success and warrant further investigation in large well-designed clinical trials.

## Electronic supplementary material

Below is the link to the electronic supplementary material.

Supplementary Material 1

Supplementary Material 2

Supplementary Material 3

## Data Availability

The data underlying this article are available in the article and its online supplementary material.
