# Supplementary material for: Clinical outcomes following endometrial receptivity assessment-guided personalized euploid embryo transfer in patients with previous implantation failures
Source: Sci Rep. 2025 May 15;15:16967. doi: 10.1038/s41598-025-01056-5 (PMC12081615; doi:10.1038/s41598-025-01056-5)
Supplement: Supplementary file 1 — Supplementary Material 1 [file 41598_2025_1056_MOESM1_ESM.docx]

| **Study type** | **Year** | **Title** | **Author(s)** | **Journal** | **Sample size** | **Main findings** |
| --- | --- | --- | --- | --- | --- | --- |
| Meta-analysis | 2022 | The Clinical Efficacy of Personalized Embryo Transfer Guided by the Endometrial Receptivity Array/Analysis on IVF/ICSI Outcomes: A Systematic Review and Meta-Analysis. | Liu Z., *et al.* | Front Physiol. 2022;27;13:841437 | 11 studies enrolled (1 RCT). Meta-analysis in good prognosis (ERA versus Non  -ERA) and RIF patients using ERA (Receptive versus non-receptive). | In good prognosis patients, OPR/LBR in non-ERA patients was lower than in ERA patients, although not statistically significant (39.5 vs 53.7%, OR 1.28, P = 0.49, 95% CI 0.92–1.77, I^2^ = 0%).  In RIF patients, there were no publications comparing ERA to non-ERA patients; however clinical outcomes in patients using ERA, were similar to those in good prognosis patients (with and without ERA: 48.7% vs 43.2%). OPR/LBR of patients with RIF undergoing pET who had non-receptive ERA increased to a level comparable to those undergoing sET with receptive ERA (40.7 vs 49.6%). |
|  | 2023 | Endometrial receptivity array before frozen embryo transfer cycles: a systematic review and meta-analysis. | Arian S., *et al.* | Fertil Steril. 2023;119(2):229-238 | Included 8 studies (N=2784 patients; 831 with ERA and 1953 without ERA). | OPR/LBR for ERA group not significantly differ from the non-ERA group (OR, 1.38; 95% CI, 0.79-2.41; I^2^ 83.0%), nor was a difference observed in subgroup analyses based on the number of previous failed ETs. The rates of implantation, biochemical pregnancy, clinical pregnancy, and miscarriages were also comparable between ERA and non-ERA groups. |
|  | 2023 | Personalized embryo transfer guided by endometrial receptivity analysis: a systematic review with meta-analysis. | Glujovsky D., *et al.* | Hum Reprod. 2023;38(7):1305-1317 | 2 RCT and 33 cohort studies comparing pET guided by TER vs standard embryo transfer (sET) in different subgroups undergoing ART. | ERA used in 85% of studies and TER in 15%. 2 RCT and 4 cohort studies compared ERA-guided pET vs sET in women with no history of RIF. No significant differences were found in LBR and CPR. In women with RIF, low CoE suggests that pET might improve CPR (OR 2.50, 95% CI 1.42-4.40). |
|  | 2023 | Comparative effectiveness and safety of 36 therapies or interventions for pregnancy outcomes with recurrent implantation failure: a systematic review and network meta-analysis. | He Y., *et al.* | J Assist Reprod Genet. 2023;40(10):2343-2356 | Meta-analysis of 154 clinical studies (29,906 RIF patients) analyzing different treatments such as AH, Acupuncture, Atosiban, ERA, G-CSF, GH, HP+EI, HA, Intralipid, PBMC, PGT-A, sequential embryo transfer (sET*); sET* hCG, and others. | Analysis indicated that ERA could improve IR (2.39, 1.52–3.72), CPR (1.71, 1.16–2.52), and LBR (3.15, 1.38–4.47). ERA rank is among the top five treatments that improve pregnancy outcomes in patients with RIF. |
| RCT | 2020 | A 5-year multicentre randomized controlled trial comparing personalized, frozen and fresh blastocyst transfer in IVF. | Simon C., *et al.* | Reprod. BioMed Online, 2020; 41(3):402-415 | N=569 patients  458 patients randomized to pET (guided by ERA), FET or fresh ET. | ITT: cumulative PR was significantly higher in the pET (93.6%) vs FET (79.7%) (P = 0.0005) and fresh ET (80.7%) (P= 0.0013). PP: at first ET: LBR was 56.2% in pET vs 42.4% in FET (P = 0.09), and 45.7% in fresh ET (P = 0.17); PR were 72.5% vs 54.3% (P = 0.01) and 58.5% (P = 0.05), respectively; IR was 57.3% vs 43.2% (P = 0.03), and 38.6% (P = 0.004), respectively. Cumulative LBR after 12 months was 71.2% vs 55.4% (P = 0.04), and 48.9% (P = 0.003), respectively. Obstetrical outcomes, type of delivery and neonatal outcomes were similar in all groups. |
|  | 2022 | Effect of Timing by Endometrial Receptivity Testing vs Standard Timing of Frozen Embryo Transfer on Live Birth in Patients Undergoing In Vitro Fertilization: A Randomized Clinical Trial. | Doyle N., et al. | JAMA, 328(21), 2117–2125 | N= 767  381 patients in the Intervention group (Receptivity-timed FET) and 386 patients in the control group (standard FET). | LBR occurred in 58.5% of transfers (223/381) in the intervention group vs 61.9% (239/ 386) in the control group (difference, -3.4% [95% CI, -10.3% to 3.5%]; rate ratio [RR], 0.95 [95% CI, 0.79 to 1.13]; P = .38). No significant differences in the intervention vs the control group for secondary outcomes, including biochemical pregnancy rate (77.2% vs 79.5%, respectively; difference, -2.3% [95% CI, -8.2% to 3.5%]; RR, 0.97 [95% CI, 0.83 to 1.14]; P = .48) and CPR (68.8% vs 72.8%, respectively; difference, -4.0% [95% CI, -10.4% to 2.4%]; RR, 0.94 [95% CI, 0.80 to 1.12]; P = 0.25). |
|  | 2024 | Assessment of the Role of Endometrial  Receptivity Analysis in Enhancing Assisted  Reproductive Technology Outcomes for  Advanced-Age Patients | Barbakadze T., *et al.* | Cureus 2024;16(6):e62949 | RCT of 320 patients in 3 arms: study group (ERA+PGTA, 35-45 years old) , control group 1  (PGT-A, 35-45 years old) and control group 2 (PGT-A+ERA <35 years old) | ERA may improve implantation and pregnancy outcomes in advanced-age  patients, particularly those with RIFs. The pregnancy rate was significantly higher in the study group (77.9%), compared to control group 1 (57.6%) (p=0.0007), and no significant difference compared to control group 2 (77.3%) (p=0.94). IR was also higher study group versus control group 1 (p=0.0009), but not vs control group 2. The live birth rate was also higher in the study group (71.3%), compared to control group 1 (39.4%) (p<0.0001). There were no significant differences between the study group and control group 2 (65.9%, p=0.50). |
| Prospective Non RCT | 2013 | [The endometrial receptivity array for diagnosis and personalized embryo transfer as a treatment for patients with repeated implantation failure.](https://www.ncbi.nlm.nih.gov/pubmed/23756099) | Ruiz-Alonso M., *et al.* | Fertil. Steril. 2013; 100(3):818-24 | RIF group: N=85  Control group: N=25. | WOI was displaced in 25.9% of patients in the RIF group vs 12% in the control group.  RIF patients after pET reached a 51.7% PR and a 38.5% IR, similar to good prognosis patients. |
|  | 2021 | Role of endometrial receptivity array for implantation failure in in-vitro fertilization & intracytoplasmic sperm injection. | Nafees R., *et al.* | Biomedica 2021;37(4):220-226. | Patients with ≥ 1 IF (N=16). | Displaced WOI in 25% of patients. PR of 75%. |
|  | 2021 | Routine endometrial receptivity array in first embryo transfer cycles does not improve live birth rate. | Riestenberg C., *et al.* | Fertil. Steril 2021; 115(4):1001-1006. | A total of 228 single euploid FET cycles. ERA group N=147  Standard ET group N=81. | ERA was receptive in 60/147 (40.8%) and nonreceptive in 87/147 (59.2%) of patients. Nonreceptive ERAs were pre-receptive in 93.1% of cases. LBR did not differ between FET with standard timing and ERA/pET, 45/81 (56.6%) and 83/147 (56.5%), respectively. Comment: Unselected patient population. |
| Retrospective | 2014 | [What a difference two days make: "personalized" embryo transfer (pET) paradigm: a case report and pilot study.](https://www.ncbi.nlm.nih.gov/pubmed/24737781) | Ruiz-Alonso M., *et al*. | Hum. Reprod. 2014; 29(6):1244-7. | Case report and case Series  with 1-6 failed transfers (N= 17). | Case report: clinical case of successful pET after 7 previous failed IVF attempts.  Case series: After pET patients reached a 60% PR vs 19% PR after ET in a non-receptive endometrium diagnosed by ERA. |
|  | 2015 | Endometrial receptivity array: Clinical application. | Mahajan N. | J. Hum. Reprod. Sci. 2015; 8(3):121-9. | RIF group (N= 80)  Control group (N=93). | In the Indian RIF population, WOI displacement was 27.5% vs 15% in the control non-RIF group (P = 0.04).  Both groups have ERA guided pET, reaching similar outcomes: RIF: 42.4% OPR and 33% IR vs Non-RIF: OPR 56% and IR 39% (p>0.1). ERA improved reproductive performance in RIF patients. |
|  | 2017 | Efficacy of the endometrial receptivity array for repeated implantation failure in Japan: A retrospective, two-centers study. | Hashimoto T., *et al*. | Reprod Med Biol. 2017; 16(3):290-296. | RIF group (N=50)  Receptive versus non-receptive. | In the Japanese RIF population, WOI displacement occurred in 24% of patients.  RIF patients with displaced or non-displaced WOI reached similar outcomes after pET: 35.3% PR in receptive patients vs 50% in non-receptive patients (P=0.9). |
|  | 2017 | [Window of implantation transcriptomic stratification reveals different endometrial subsignatures associated with live birth and biochemical pregnancy.](https://www.ncbi.nlm.nih.gov/pubmed/28863933) | Diaz-Gimeno P., *et al.* | Fertil. Steril. 2017; 108(4):703-710.e3. | Fertile donors (N=79)  ERA patients (N=771). | OPR ranged from 76.9% and 80% in the late pre-receptive and receptive stages, respectively, vs 33.3% when ET performed in the late-receptive stage. BPR was 7.7% and 6.6% in the late pre-receptive and receptive stages, respectively, but 50% when ET performed in the late-receptive stage. |
|  | 2018 | The role of the endometrial receptivity array (ERA) in patients who have failed euploid embryo transfers. | Tan J., *et al.* | J. Assist. Reprod. Genet. 2018; 35(4): 683-92. | ERA group with ≥1 previously failed euploid transfer (N=88).  pET vs Standard ET. | WOI displacement in 22.5% of patients. RIF patients with displaced or non-displaced WOI reached similar outcomes after pET. IR and OPR were higher in euploid pET vs euploid standard ET patients (76.5 vs 53.8% and 64.7 vs 42.3%, respectively) although not statistically significant. |
|  | 2018 | Does the endometrial receptivity array really provide personalized embryo transfer? | Bassil R., *et al.* | J. Assist. Reprod. Genet. 2018; 35(7):1301-1305. | ERA group with 0-2 failed transfers (N=53)  Standard ET group with 0-2 failed transfers (N=503). | WOI displacement occurred in 64.15% of ERA patients.  No statistically significant differences in PR between the pET and ET groups (39% vs 35.2%). |
|  | 2018 | Window of implantation is significantly displaced in patients with adenomyosis with previous implantation failure as determined by endometrial receptivity assay. | Mahajan N., *et al.* | J.Human. Reprod. Sci. 2018; 11(4):353. | Adenomyosis group (N=36)  Control group (N=338). | In adenomyosis, WOI was significantly displaced (47.2%) vs controls (21.6%) (p < 0.001). Risk ratio 2:1.  The incidence of RIF in adenomyosis was 66.6% versus 34.9% in controls (P < 0.001).  PR after pET in adenomyosis group was 62.5%. |
|  | 2019 | Personalized Embryo Transfer Helps in Improving In vitro Fertilization/ICSI Outcomes in Patients with Recurrent Implantation Failure. | Patel JA., *et al.* | J. Hum. Reprod. Sci. 2019; 12(1):59-66. | RIF group (N= 248)  Receptive vs non-receptive. | WOI displacement in 17.7% of RIF patients.  RIF patients with displaced or non-displaced WOI reached similar outcomes after pET.  OPR was 41.7% vs 42.9% (P=0.93).  pET guided by ERA in patients of RIF with displaced WOI improves IR and OPR. |
|  | 2019 | Endometrial Receptivity Analysis - a tool to increase an implantation rate in assisted reproduction. | Hromadová L., *et al*. | Ceska Gynekol. 2019; 84(3): 177-183. | ERA group (N=85). | WOI displacement in 36.5% of patients.  PR after pET in non-receptive patients was 69.2%. This study supports the identification of the WOI. |
|  | 2019 | What is the clinical impact of the endometrial receptivity array in PGT-A and oocyte donation cycles? | Neves AR., *et al.* | J. Assist. Reprod. Genet. 2019; 36: 1901-1908 | pET group with ≥1 previously failed euploid ET (N=24) or with ≥2 previously failed donor transfers (N=32)  Standard ET group with ≥1 previously failed euploid ET (N=119) or ≥2 previously failed donor transfer (N=158). | WOI was displacement was 41.1%.  After euploid ET no differences in pET vs ET groups were found (IR 55.6% vs 65% and PR 58.3% vs 70.6%).  Significant lower PR (34.4% vs 65.2%; P = 0.001) in donor pET group vs donor standard ET group.  RIF patients with displaced or non-displaced WOI reached similar outcomes after pET in both euploid and donor arms. |
|  | 2020 | Evaluation of the endometrial receptivity assay and the preimplantation genetic test for aneuploidy in overcoming recurrent implantation failure. | Cozzolino M., *et al.* | J. Assist. Reprod. Genet. 2020; 37(12):2989-2997. | Moderate RIF group: (N=2110)  Severe RIF group (N= 488). | PGT-A, ERA, or ERA+PGT-A vs the control group with no testing. Patients with euploid ET in the moderate RIF group had higher IR and OPR than those without PGT-A. ERA did not significantly improve clinical outcomes in either group. |
|  | 2020 | Comparing endometrial receptivity array to histologic dating of the endometrium in women with a history of implantation failure. | Cohen AM., et al | Syst. Biol. Reprod. Med. 2020; 66(6):347-354. | RIF group (N=97). | WOI displaced in 47.4% of patients.  Concordance between ERA and histological dating was 40.0%.  RIF patients with displaced (22.5%) or non-displaced WOI (26.7%) reached similar CPR after pET, P = 0.66. |
|  | 2020 | Does personalized embryo transfer based on era improve the outcomes in patients with thin endometrium and RIF in Self Versus Donor Programme? | Selvaraj P., *et al*. | J. Gyneccol. Res.h and Obstetrics. 2020;6(3): 076-080. | RIF self-oocyte ERA (N=179)  RIF self-non-ERA (N=181)  RIF donation ERA (N=180)  RIF donation non-ERA (N=182). | Study done in patients with thin endometrium. Displaced WOI in 35-39% of patients.  Clinical outcomes were not statistically different between patients with self-oocytes with/without ERA (due to embryo factor) but significantly higher in ovum donation patients with ERA (CPR 59.4%) than without ERA (CPR 43.4%) |
|  | 2021 | [Clinical utility of the endometrial receptivity analysis in women with prior failed transfers.](https://pubmed.ncbi.nlm.nih.gov/33454901/) | Eisman LE., *et al.* | J. Assist. Reprod. Genet. 2021; 38(3):645-650. | ERA group with ≥1 previously failed ET (N=131)  ERA group without previous failures (Control group) (N=91). | WOI displaced in 45% of patients with ≥1 failed ET, 40% with ≥3 previously failed ET and 52% of control patients.  Pregnancy outcomes did not differ between women with ≥ 1 prior failed ET and controls. Women with ≥ 3 prior failed ETs had lower OGP/LBR (28% vs 54%, P = 0.046) assuming other factors beyond displaced WOI could be implied in cases of failure. |
|  | 2021 | Evaluation of Pregnancy Outcomes of Vitrified-Warmed Blastocyst Transfer before and after Endometrial Receptivity Analysis in Identical Patients with Recurrent Implantation Failure. | Kasahara Y., *et al*. | [Fertility & Reprod](https://www.worldscientific.com/worldscinet/fandr). 2020; [3(2):35-41](https://www.worldscientific.com/toc/fandr/03/02). | RIF group (N=94). | Displaced WOI in 52.1%. of patients. A comparison of previous ET and pET in RIF patients demonstrated a significant increase in PR for pET per patient and cycle (5.3% vs 62.8%, 4.4% vs 47.9%, respectively). PR and IR at the first pET were significantly higher in patients with displaced vs non-displaced WOI. |
|  | 2021 | The use of propensity score matching to assess the benefit of the endometrial receptivity analysis in frozen embryo transfers. | Bergin K., *et al*. | Fertil. Steril. 2021; 116(2):396-403. | ERA group (N=133)  Non-ERA group (N= 353). | LBR for the ERA group (49.62%), and matched non-ERA group (54.96%), (odds ratio 0.8074; 95% confidence interval, 0.5424-1.2018) were not significantly different, nor was a difference seen in subanalyses based on prior number of FETs or receptivity status. |
|  | 2021 | Evaluation of embryo aneuploidy (PGT-A)  and endometrial receptivity (ERA) testing in patients with recurrent implantation  failure in ICSI cycles. | Fodina V., *et al.* | Gynecol. Endocrinol. 2021; 37(S1):17-20. | 253 RIF patients. 4 groups:  I: FET (72)  II: FET+PGT-A (87)  III: FET+PGT-A+ERA (72)  IV: FET+ ERA (22). | Group II had significantly higher change of achieving biochemical and clinical pregnancy than Group I. Groups III and IV were similar to Group I. |
|  | 2021 | Correlation between plasmatic progesterone, endometrial receptivity genetic assay and implantation rates in frozen-thawed transferred euploid embryos. A multivariate analysis. | Barrenetxea G., *et al.* | Eur. J. Obstet. Gynecol. Reprod. Biol. 2021;263:192-197. | N=104. | ERA was non-receptive in 45,31% of patients.  Overall, LBR after FET of euploid embryos was 62,35%. The odds of pregnancy were higher when ERA was performed before the first ET (93,10% vs 46,43%; OR = 15,58;95% CI 3,38-71,89). Overall, OPR showed a favourable trend after a “non-receptive” endometrium was diagnosed and thus, a modified (pET) preparation was performed (70,00% vs 55,56%; OR = 1,87; 95% CI 0,76-4,57). |
|  | 2022 | Do clinical outcomes differ for day-5 versus day-6 single embryo transfers controlled for endometrial factor? | Stankewicz T., *et al*. | Reprod. Biomed. Online. 2021 Nov 18:S1472-6483(21)00581-2. | Day 5 blastocysts: N=183  Day 6 blastocysts: N=77. | Clinical outcomes were similar when transferring day-5 blastocysts vs day-6 blastocysts: PR was 75.4% and 70.1% (P = 0.465); IR was 67.8% and 63.6% (P = 0.476); and OPR 57.9% and 58.4% (P = 0.728). |
|  | 2022 | Role of endometrial receptivity array in recurrent implantation failure. | Samadhiya R., *et al.* | Fertill. Scii and Res 2021;8(2): 180-18. | RIF patients (N=34). | WOI displacement in 38.2% of patients.  PR and IR after pET in non-receptive group reached 50% and 45.5%, similar to the 55.4% obtained in general patients (non-RIF). |
|  | 2022 | [Comparison of the Effectiveness of Endometrial Receptivity Analysis (ERA) to Guide Personalized Embryo Transfer with Conventional Frozen Embryo Transfer in 281 Chinese Women with Recurrent Implantation Failure](javascript:void(0)). | Jia Y., *et al.* | Med. Sci. Monit: Intern. Med.l J. of Experiment. and Clinic. Res. 2022;28:e935634-1. | RIF ERA group (N=140)  RIF Non-ERA group (N=141). | ERA identified 35% of samples as receptive and 65% as non-receptive in the ERA group.  Higher CPR and IR were found in the ERA group than in the non-ERA group (CPR with ERA 50% vs 24.8% without ERA and IR with ERA 41.7% vs 18.8% without ERA; P<0.01), while no significant differences were detected between the groups in terms of miscarriage rates (P>0.05). |
|  | 2022 | Personalized Embryo Transfer Outcomes in Recurrent Implantation Failure Patients Following Endometrial Receptivity Array with Pre-Implantation Genetic Testing. | Amin J., *et al.* | Cureus. 2022; 14(6): e26248. DOI 10.7759/cureus.26248. | 291 RIF patients were categorized into Group I (patients without ERA group) and Group II (ERA study group) and subclassified into receptive and nonreceptive ERA groups. | CPR (23% and 48.4%) and IR (4.1% and 50.6%) were higher in the ERA group vs the Non-ERA group. CPR was (64% and 65%) and IR was (65% and 74%) in receptive and non-receptive ERA, respectively. They concluded: “Endometrial receptivity assessment is a highly beneficial method to assess the genetic expression of the endometrium and embryo transfer timing”, ERA results-guided pET improved IR and reproductive outcomes in RIF patients. |
|  | 2022 | Live birth after transfer of a single euploid vitrified-warmed blastocyst according to standard timing vs timing as recommended by endometrial receptivity analysis. | Doyle N., *et al.* | Fertil Steril. 2022;118(2):314-321. | Patients with/without ERA before euploid single FET.  N=307 ERA guided FETs and  N=2284 standard protocol FETs. | 125 patients (40.7%) were ERA receptive, and 182 (59.3%) were ERA non-receptive. No statistically significant differences in LBR in patients with ERA-receptive vs ERA-nonreceptive results (48.8% and 41.7%, respectively; adjusted odds ratio 1.17; 95% CI, 0.97-1.40). No statistically significant differences in LBR in patients with or without ERA testing results before FET (44.6% and 51.3%, respectively; adjusted odds ratio 0.87; 95% CI, 0.73-1.04). |
|  | 2022 | Use of the endometrial receptivity array to guide personalized embryo transfer after a failed transfer attempt was associated with a lower cumulative and per transfer live birth rate during donor and autologous cycles. | Cozzolino M., *et al*. | Fertil. Steril. 2022;118(4):724-736. | Patients with a single previous failed ET; yielded 3,239 autologous transfers and 2,133 donor transfers. | During both autologous or donor transfers, the LBR and cumulative LBR were higher in FET and fsET than in pET groups, even with euploid transfers. Logistic regression analysis, considering possible confounders, indicated that patients receiving pET had poorer outcomes than those undergoing FET and fsET in autologous and donor cycles. IR, PR, and CPR were lower in patients undergoing pET. |
|  | 2022 | Identifying women with a narrow window of embryo implantation using the endometrial receptivity assay. | Rose B. | Internat. J.ournal of Clinic. Obstet. and Gynaecol. 2022;6(3):52-54. | 24 patients who had failed to achieve pregnancy after the transfer of multiple good-quality embryos. | FET executed after approximately 144 hours of progesterone exposure to estrogen prepared endometria. The ERA recommended a change in the duration of progesterone exposure in 70.8% of women evaluated. Changes in progesterone duration based on ERA led to an IR of 50%, CPR of 76.5% and LBR of 64.7%. This compares to an IR of 3.4% and LBR of 1.1% for ETs before ERA. |
|  | 2022 | Effectiveness comparison between endometrial receptivity array, immune profiling and the combination in treating patients with multiple implantation failure. | Jia Y., *et al*. | Am. J. l of Reprod. Immunol. (New York, N.Y.: 1989), 87(3), e13513. | N=172 with ≥2IF  Four groups:  No treatment  Immune profiling  ERA  ERA+Immune profiling. | The overall incidence rate of the displaced window of implantation (WOI) and endometrial immune dysregulations were 84.9% and 75.3%, respectively. Implantation rate was significantly higher in the 'ERA + Immune Profiling' group than the 'no treatment' group (P = .007). Clinical pregnancy rate was somewhat improved in the three treatment groups but with a borderline significance (P = .071). After controlling for other confounders, 'ERA + Immune Profiling' treatment was associated with a higher pregnancy rate [aOR (95%CI) = 3.412 (1.387-8.395), P = .008]. There was no association between endometrial immune profiling and ERA phases. |
|  | 2023 | Assessing endometrial receptivity after recurrent implantation failure in euploid embryo transfer: a retrospective study in private clinic. | Luján-Irastorza JE. | J. of Reprod. 2023; 2(1). | 37 RIF patients after transfer of good quality, euploid (PGT-A) blastocysts. | Of the 100% of the patients, 43% of patients presented a normal WOI. 61.5% showed a higher prevalence of natural killer alterations and 42.8% thrombophilias. Patients with altered endometrial receptivity, presented with a cumulative IR higher than 70%. |
|  | 2023 | Clinical outcomes of personalized blastocyst embryo transfer after endometrial receptivity analysis: A multicenter, retrospective cohort study. | Takeshige Y., *et al*. | Reproductive medicine and biology, 22(1), e12550. | N=861 women with ERA between 2018 and 2020.  Receptive vs non-receptive. | Non-receptivity in 41% (353/861) of patients.  CPR, miscarriage, and LBR for personalized blastocyst ET were 44.5% (226/508), 26.1% (59/226), and 26.8% (136/508) for receptive patients, and 43.1% (152/353), 28.3% (43/152), and 28.9% (102/353) for non-receptive patients (all not statistically significant).  Increased patient age, smoking, and longer duration of infertility significantly negatively associated with receptivity, whereas a history of delivery significantly positively associated with receptivity. |
|  | 2024 | Exploring the effectiveness of endometrial receptivity array and immune profiling in patients with multiple implantation failure: A retrospective cohort study based on propensity score matching. | Jia Y., *et al.* | J. Reprod. Immunol. . online February 17, 2024. | N=1429 with ≥2 consecutive IF.  Four groups:  No test  Immune profiling  ERA  ERA+Immune profiling. | Overall incidence of displaced WOI and endometrial immune dysregulation was 75.14% and 79.29%, respectively.  'ERA' and 'ERA + Immune Profiling' groups demonstrated significantly higher rates of biochemical, CPR, OPR, and IR compared to the 'No test' group (p < 0.01). The 'Immune Profiling' group showed a higher IR than 'No test' group (p < 0.05). When comparing three test groups, the 'ERA + Immune Profiling' group exhibited notably higher rates of CPR and OPR than the 'Immune Profiling' group (p < 0.017). |
| Case report | 2014 | Live birth after embryo transfer in an unresponsive thin endometrium. | Cruz F. &  Bellver J. | Gynecol. Endocrinol. 2014; 30(7):481-4. | Case report. | WOI found in an endometrium with a thickness of 3.5 mm with subsequent LB achieved after pET. |
|  | 2018 | Different Endometrial Receptivity in Each Hemiuterus of a Woman with Uterus Didelphys and Previous Failed Embryo Transfers. | Carranza F., *et al.* | J Hum Reprod Sci. 2018; 11(3):297-299. | Case report. | ERA showed receptivity in the right-sided hemiuterus while the left-sided hemiuterus was non-receptive. LB achieved after pET in the right-sided hemiuterus. |
|  | 2019 | Why results of endometrial receptivity assay testing should not be discounted in recurrent implantation failure? | Simrandeep K., *et al.* | Onco Fertility Journal. 2019; 2(1): 46-49. | Cases report (N=3). | 3 severe cases of had a previous ERA performed at a different center, pET was not followed, resulting in failures. Once pET was implemented, successful clinical pregnancy was achieved in all patients. |
|  | 2019 | The Reproductive Outcomes for the Infertile Patients with Recurrent Implantation Failures May Be Improved by Endometrial Receptivity Array Test. | Ota T., et al. | J.l of Medical Cases. 2019; 10(5), 138-140. | Case report. | 34 year-old patient achieved pregnancy with ERA guided pET by after 11 previous failed attempts (4 biochemical miscarriages, 1 clinical miscarriage and 6 negative pregnancies) |
|  | 2021 | Older Women's Reproductive Outcomes May Not Be Improved by the Endometrial Receptivity Analysis Test: A Case Report. | Fujishima R., *et al*. | Cureus, 13(12), e20735. | Case report. | Case of a 39-year-old woman with refractory RIF using ERA. This research showed the limitation of ERA in patients with RIF. This will save resources and time before any test or investigation for the diagnosis and management of such patients. |
|  | 2024 | A Case Report on Endometrial Receptivity Array  Test for Infertile Patient to Enhance  Reproductive Outcomes. | Khan, *et al*, | Cureus 16(2): e55059. | Case report. | A 34-year-old woman with unexplained RIF. 5 IUI and 4 IVF cycles were unsuccessful.  WOI determined the ET timing after ERA. The patient's clinical pregnancy was successful. This case focused on the potential of the ERA test to improve reproductive outcomes. |
|  | 2024 | Endometrial Receptivity Array (ERA) Test in a 32-Year-Old Female with Refractory Infertility: A Case Report. | Imoto S., *et al*. | Cureus 16(3): e55703. | Case report | An LB case for a 32-year-old patient with refractory infertility using ERA. |

**Supplementary Table 1**. Publications analysing the clinical outcomes of pET guided by ERA in different indications in IVF patients. Abbrevations – AH: assisted hatching; aOR: adjusted odds ratio; ART: assisted reproductive technologies or assisted reproduction techniques; BPR: biochemical pregnancy rate; CI: confidence interval; CoE: certainty of evidence; CPR: clinical pregnancy rate; EI: endometrial injury; ERA: endometrial receptivity analysis; ET: embryo transfer; FET: frozen embryo transfer; fsET: fresh embryo transfer; G-CSF: granulocyte-colony stimulating factor; GH: growth hormone; HA: hyaluronic acid; hCG: human chorionic gonadotropin; HP: hysteroscopy; ICSI: intracytoplasmic sperm injection; IF: implantation failure; IR: implantation rate; ITT: intention-to-treat analysis; IUI: intrauterine insemination; IVF: in vitro fertilisation; LB: live birth; LBR: live birth rate; N: sample size; OPR: ongoing pregnancy rate; OR: odds ratio; p: p-value; PBMC: peripheral blood mononuclear cell; pET: personalized embryo transfer; PGT-A: preimplantation genetic testing aneuploidy; PP: per protocol analysis; PR: pregnancy rate; RCT: randomised controlled trial; RIF: repeated or recurrent implantation failure; RR: rate ratio; sET: standard embryo transfer; sET*: sequential embryo transfer; TER: tests for endometrial receptivity; vs: versus; WOI: window of implantation.
