# Supplementary material for: Clinical outcomes following endometrial receptivity assessment-guided personalized euploid embryo transfer in patients with previous implantation failures
Source: Sci Rep. 2025 May 15;15:16967. doi: 10.1038/s41598-025-01056-5 (PMC12081615; doi:10.1038/s41598-025-01056-5)
Supplement: Supplementary file 2 — Supplementary Material 2 [file 41598_2025_1056_MOESM2_ESM.docx]

| **PGT-A Subgroup** | **ERA-guided pET** | **Standard ET** | **P-value** |
| --- | --- | --- | --- |
| Number of patients | 177 | 62 | --- |
| Implantation rate (%) | 101/177 (57.1) | 22/62 (35.5) | 0.005 |
| Pregnancy rate (%) | 113/177 (63.8) | 25/62 (40.3) | 0.002 |
| Biochemical pregnancy rate (%) | 11/113 (9.7) | 3/25 (12.0) | 0.72 |
| Clinical miscarriage rate (%) | 17/113 (15.0) | 4/25 (16.0) | 0.99 |
| Ectopic pregnancy rate (%) | 1/113 (0.9) | 0 | 0.99 |
| Ongoing pregnancy rate (%) | 84/177 (47.5) | 18/62 (29.0) | 0.02 |
| Lost to follow-up rate (%) | 3/177 (1.7) | 1/62 (1.6) | 0.99 |
| Live birth rate (%) | 81/174 (46.6) | 17/61 (27.9) | 0.02 |

**Supplementary Table 2**. Data expressed as %. Variables compared using the Chi-square or Fisher exact test. P-values indicate statistically significant differences between groups. Abbreviations – ERA: endometrial receptivity analysis; ET: embryo transfer; pET: personalized embryo transfer; PGT-A: Preimplantation genetic testing for aneuploidy.
