# Supplementary material for: Clinical outcomes following endometrial receptivity assessment-guided personalized euploid embryo transfer in patients with previous implantation failures
Source: Sci Rep. 2025 May 15;15:16967. doi: 10.1038/s41598-025-01056-5 (PMC12081615; doi:10.1038/s41598-025-01056-5)
Supplement: Supplementary file 3 — Supplementary Material 3 [file 41598_2025_1056_MOESM3_ESM.docx]

|  | **aOR Ratio** | **95% CI** | **P-value** |
| --- | --- | --- | --- |
| Age | 1.05 | 0.98 - 1.14 | 0.17 |
| BMI | 0.91 | 0.84 - 0.99 | 0.04 |
| Previous failed attempts | 1.14 | 0.96 - 1.36 | 0.14 |
| Number of embryos transferred | 1.58 | 0.68 - 3.67 | 0.29 |
| Embryo quality | 1.13 | 0.62 - 2.06 | 0.69 |
| Transfer by ERA recommendation | 2.83 | 1.49 - 5.57 | 0.002 |

**Supplementary Table 3**. Adjusted odds ratios and 95% confidence intervals shown for each coefficient. Body-mass index (BMI) represents weight in kilograms divided by the square of the height in meters. Abbreviations – aOR: adjusted odds-ratio; CI: confidence interval; ERA: endometrial receptivity analysis; PGT-A: Preimplantation genetic testing for aneuploidy.
